# The effectiveness and safety of Iranian herbal medicines for treatment of premenstrual syndrome: A systematic review

**Published:** 2018

**Authors:** Nahid Maleki-Saghooni, Fatemeh Zahra Karimi, Zahra Behboodi Moghadam, Khadigeh Mirzaii Najmabadi

**Affiliations:** 1 *Student Research Committee, Department of Midwifery and Reproductive Health, Nursing and Midwifery School, Mashhad University of Medical Science, Mashhad, Iran*; 2 *Department of Midwifery and Reproductive Health, Nursing and Midwifery School, Mashhad University of Medical Sciences, Mashhad, Iran*; 3 *Department of Midwifery and Reproductive Health, Nursing and Midwifery School, Tehran University of Medical Sciences, Tehran, Iran*

**Keywords:** Herbal medicine, Premenstrual syndrome, Systematic review

## Abstract

**Objective:**

Premenstrual syndrome (PMS) is one of the most common problems among women of reproductive age. The popularity of complementary/alternative therapies has grown in recent years, and these treatments have been more commonly used by women (48.9%) than men (37.8%). The aim of this systematic review was to assess effectiveness and safety of Iranian herbal medicines for treatment of premenstrual syndrome.

**Methods:**

PubMed, Scopus, Cochrane, and Google Scholar were searched along with SID, Magiran and Irandoc up to Dec 2017.

Inclusion criteria consist of Iranian, published, randomized controlled trials (RCTs) using Iranian herbal medicine for treatment of reproductive age women with PMS. Eventually Eighteen RCTs met the inclusion criteria.

**Results:**

Overall, studies have shown that* Vitex agnuscastus, Hypericum perforatum, Matricaria chamomilla, saffron, Curcumin*, *Melissa officinalis*, *Zataria multiflora,* *Wheat Germ Extract, Echinophora platyloba, Foeniculum vulgare, Valerian root extract, Citrus sinensis, Zingiber officinale *and *Flax seed* might alleviate symptoms of PMS.

**Conclusion:**

This research demonstrated efficacy and safety of Iranian herbal medicines in alleviating PMS. Therefore, herbal medicine can be regarded as an alternative treatment for women suffering from PMS.

## Introduction

Premenstrual syndrome (PMS) is one of the most common problems among women of reproductive age (Lethaby et al., 2012 ▶, Taavoni et al., 2014 ▶). This syndrome was first characterized in 1931 by Frank and Horney (Filho et al., 2011 ▶). PMS refers to a set of psychological and physical symptoms such as depression, hopelessness, anxiety, mental tension, emotional disturbances, violence , face blushing, low desire to do routine jobs, distraction, reduction of energy, rapid fatigue, changes in diet, sleep disturbances, abdominal pain, breast tenderness, headache, muscles cramp, fatigue, edema and weight gain which are experienced by a few women in the late luteal phase (Lentz and Henshaw 2007 ▶, Hankinson et al., 2010 ▶). About 80–90% of women experience PMS before menstrual bleeding. Premenstrual dysphoric disorder (PMDD) is known as extreme sort of PMS which affects the person's daily activities. It is recognized in 3–5% of women ( Stutz et al.,2011 ▶, Tolossa and Bekele 2014 ▶). In Iran, the prevalence of PMS is 67-78.4% (Soltan Ahmadi et al., 2007 ▶, kiani et al., 2009 ▶). Moreover, PMS is more common among white women, smokers, obese, and youthful women (Masho et al., 2005 ▶). This syndrome has a multifactorial etiology which is not completely characterized. At first, PMS is mainly associated with diminished levels of progesterone in the luteal phase (Speroff and Fritz 2010 ▶). Different etiologies described for PMS include abnormal neurotransmitter reactions to ordinary ovarian functions, hormonal imbalances, sodium retention, and/or nutritional deficiencies (Dickerson et al., 2003 ▶). The manifestations of PMS may be adequately serious to disturb women’s regular functioning, quality of life, social relationships and can lead to increased rates of suicides, accidents, joblessness, work and school absenteeism, and poor scholastic execution, (Mishell 2005 ▶, Lowin et al., 2011 ▶). Besides, reproductive health problems such as child abuse and domestic violence have also been observed in families with individuals experiencing PMS. Therefore, this syndrome not only influences the individual herself, but also affects the family and even the society (Pinar et al., 2011 ▶). Based on the literature, many different pharmacological and non-pharmacological treatments have been recommended as possible therapies for women with PMS (Kessel, 2000 ▶; Moline and Zendell, 2000 ▶; Stevinson and Ernst, 2001 ▶; Douglas, 2002 ▶; Girman et al., 2003 ▶; Rapkin, 2003 ▶; Wong et al., 2010 ▶; Hur et al., 2011 ▶; Vishnupriya and Rajarajeswaram, 2011 ▶; Zamani et al., 2012 ▶, Samadi et al., 2013 ▶; Delaram, 2014 ▶; Saki et al., 2015 ▶) because of uncertainty of its pathogenesis, the extensive variety of its signs, and a large placebo impact. For example, selective serotonin reuptake inhibitors (SSRIs) have been evaluated as a first-line therapy for PMS (Steiner et al., 2006 ▶). Despite this fact that PMS symptoms relief is ambiguous, it is rational to propose healthy lifestyle changes which may have general advantages (Douglas 2002 ▶, Girman et al. 2003 ▶, Rapkin 2003 ▶).

 For long period of time, plants have been assumed as important treatments for numerous ailments, particularly in Eastern nations (Fallah Huseini et al., 2006 ▶). Medicine has always played a significant role in Iranian culture and civilization. Due to its long history and hundreds of publications, Iranian traditional medicine is among the oldest and richest alternative medicines (Emtiazi, 2012 ▶ ). Since Iran has a wide variety of medicinal plants and also as Iranians are interested in using them, herbal medicine has become increasingly popular among Iranian population (Ghasemi dehkordi et al, 2003 ▶). Also, these days, complementary medicine and herbal products is more commonly used by women (48.9%) than men (37.8%) (Amasha et al. 2017 ▶). Therefore, Iranian researchers have investigated the effects of various native herbal medicines for treatment of PMS (Domoney et al., 2003 ▶); however, there is no evidence indicating the safety of different herbs or reporting their adverse events, so a review study is required for access to reliable and evidence-based information (Younesi, 2014 ▶). To the best of our knowledge, the efficacy of Iranian herbal medicine in treatment of PMS has not been systematically assessed. This systematic review aimed to examine the effectiveness and safety of Iranian herbal medicines for treatment of PMS based on the articles published in this field.

## Materials and Methods

A systematic literature review was conducted utilizing the electronic databases such as Pub Med, Scopus, Cochrane and Google Scholar as well as Persian databases such as SID, Magiran and Irandoc up to Dec 2017 using appropriate keywords including “Premenstrual Syndrome or PMS“AND “alternative medicine, Complementary Therapies, medicinal plants, Herbal medicine, Herbal Remedies, Plant Extracts, Iranian Herbal Medicines“in the “title”, “abstract”, or “keywords”, until Dec 2017. All databases were searched for Iranian published randomized controlled trials (RCTs). Duplicate articles, or articles which were inconsequential to the investigation, were not considered in the review. Moreover, reference section of relevant trials, systematic reviews and meta-analyses were checked to recognize further trials missed by electronic search. In the process of article extraction, one of the analysts checked both the title of the article and the abstract and judged its appropriateness for inclusion.

If the selected articles met the following criteria, they were included in this review:

1) Conducted on the humans.

2) Being a randomized controlled trial (RCTs).

3) Performed by Iranian authors using Iran native plants.

4) Female participants of reproductive age with premenstrual disorders who experienced at least one symptom happening periodically during day 0 to 14 before menstruation, for at least three menstrual cycles.

5) Interventions consisted of any formulation of Iranian herbal medicine including oral preparations, decoctions, injections, and tablets. Comparison groups could comprise of a placebo or some other interventions. Evaluations of the effect of a specific herbal medicine versus another herbal medicine were also included.

6) Outcome measures included:

Primary outcomes: Change of general manifestations by Daily Symptom Record (DSR), prospective record of the impact and severity of menstrual symptoms (PRISM) calendar, shortened premenstrual assessment form (SPAF), visual analogue scale (VAS) that is a rapid and straightforward assessment and ranging from 0 (no symptoms) to 10 (unbearable) and assess following six symptoms (headache, nervousness, restlessness, depression, breast swelling and pain, bloating and tympani) (Zamani etal., 2012 ▶) and Premenstrual Syndrome Screening Tool (PSST). The following tools were considered for Secondary outcomes: 

1. Quality of life

2. Adverse events

3. Daily form of MAPMS (Mastalgia intensity Associated with PMS) 

4. Hamilton Depression Rating Scale

5. GHQ-28 questionnaire

6. The Beck Depression Inventory (BDI)

**Figure1:**
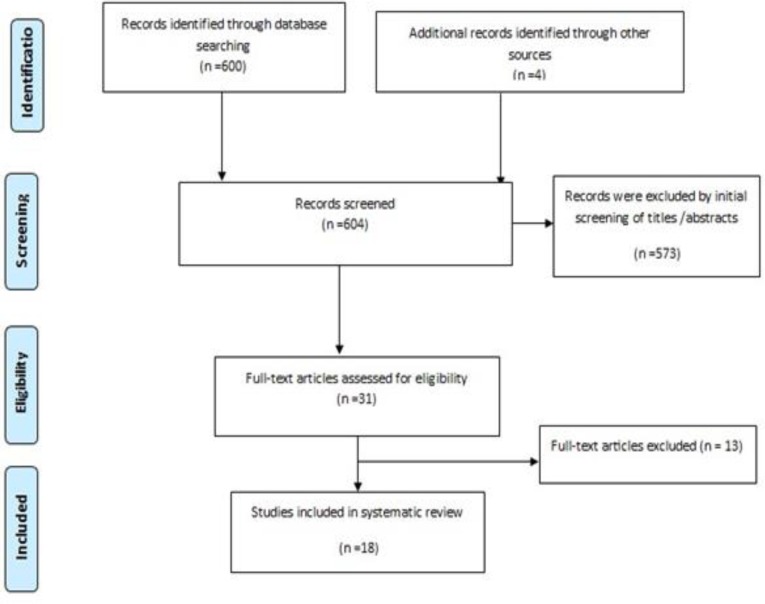
PRISMA flow diagram of article selection progress.

The process of the search and selection of RCTs is shown in Figure 1.

The articles selection and data analysis were undertaken by two researchers. Based on the search results, authors browsed the abstracts and relevant records. Full articles were retrieved for all conceivably significant trials. All articles were scrutinized to check for multiple publications on the same trials. For each study, we extracted the following data according to a pre-defined checklist: first author, year, design, study period, subjects’ age, intervention, control, number of participants in intervention and control groups, baseline comparability, dropout, tools, blinding method, adverse effect, outcomes and quality of trials which evaluated by two reviewers using Oxford Center for Evidence-Based Medicine checklist for therapeutic studies. Data were independently assessed by two authors and incongruities were settled by discussion with a third researcher. Overall, there was complete agreement between the two reviewers. The characteristics of the included studies are shown in Table 1.

**Quality assessment of the included studies**

The quality of the included studies was assessed by Oxford Center for Evidence-Based Medicine checklist for RCTs (see Table 2).

List of criteria for surveying the quality of studies consist of: 

A: Was the assignment of patients to treatments randomized?

B: Were the groups similar at the beginning of the trial?

C: Aside from the allocated treatment, were groups treated equally?

D: Were all patients who entered the trial accounted for? – And were they analyzed in the groups to which they were randomized? (1: Losses to follow-up and 2: intention-to-treat)

E: Were measures objective or were the patients and clinicians kept “blind” to treatments?

F: What were the results? (www.cebm.net/ University of Oxford).

## Results

From 604 relevant trials, 18 RCTs met the inclusion criteria. The characteristics of included studies summarized in Table 1.

**The effect of** ***Matricaria chamomila*** **(Chamomile) extract on PMS**

*Chamomile vs. Mefenamic Acid*

Sharifi et al. compared two groups treated with chamomile and mefenamic acid (MA), and a significant decrease (p<0.0001) was found in average mastalgia when comparing pre and post-intervention after first and second cycles in those treated with chamomile extract (10.5±21.7 and 13.7±20.4 percent) and among MA-treated subjects (12.1±24.7 and 13.8±24 percent). However, there was no significant difference in PMS symptom in chamomile extract and MA groups after first and second cycles (p>0.05). The side effects after two periods of treatment included menstrual bleeding in nine (20%) patients receiving chamomile (Sharifi et al., 2014).

**The effect of** ***Crocus sativus L.*** **(saffron) on PMS**

Agha-Hosseini et al. assessed the effect of saffron on the PMS using two-way repeated measures analysis of variance to assess the differences within and between groups that demonstrated a significant impact of saffron on the Total Daily Symptom Ratings (p<0.0001). In saffron group, *post-hoc* correlations demonstrated a significant change from cycle 3. A significant difference between cycles 3 and 4 was seen in the saffron group (p<0.001). The difference between the two protocols was significant at cycle 4 (t=5.92, df=48, p<0.001). In addition, one-way repeated measures analysis of variance demonstrated a significant impact of saffron on Hamilton Depression Rating Scale (p<0.0001). In saffron group, *post-hoc* correlations demonstrated a significant change from cycle 3. A significant differences between cycles 3 and 4 were seen in the saffron group (p<0.001). The difference between the two protocols was significant at cycle 4 (t=8.99, df=48, p<0.001). Six adverse effects were seen in this trial. The difference between the saffron and placebo in the recurrence of adverse impacts was not significant (Table 2). None of adverse impacts was severe. Appetite changes and headache were more common in the saffron group, yet not noteworthy (Agha‐Hosseini, Kashani et al. 2008 ▶) In another trial, Pirdadeh Beiranvand et al., using repeated measures test, showed that the difference in the severity of PMS symptoms over time in the intervention group (p<0.001) and in the control group (p=0.04) were statistically significant; also, in terms of time–group interaction, a significant difference in changes of the mean severity of PMS was shown between the two groups over time (p<0.001). Chi-square test indicated no significant differences in terms of side effects such as increased appetite, loss of appetite, sedation, nausea, headache, and euphoria between the two groups (p>0.05) (Beiranvand et al., 2015 ▶).

**Table1:** Characteristics of 18 randomized controlled trials included in systematic review.

**Outcome(s)**	**Adverse effects**	**blinding method**	**Tool** **s**	**Dropouts** ** (%)**	**Baseline comparability**	**Participants ** **control**	**Participants Intervention**	**Type of control**	**Intervention mg**	**Age (/Y)**	**Duration, (wk.)**	**Design**	**Author, Year**	**No**
Significant decrease in average mastalgia was observed between Chamomile Extract and MA-users in pre and post intervention after first and second cycles. However, no significant difference in average MAPMS symptoms was observed between chamomile Extract-users after first and second cycles Intra group difference(Wilcoxon test) p<0.0001	menstrual bleeding in nine (20%) of chamomile GI complication in (28.9%) of MA	Double blind	Daily form of MAPMS (Mastalgia intensity Associated with PMS)	26%	Yes	N=45	N=45	Mefenamic Acid 250 mg	Chamomile capsule 100 mg	18 to 35	4	RCT	Sharifi et al. (2014), Iran	1
Saffron was found to be effective in relieving Symptoms of PMS. A significant difference between cycles 3 and 4 was observed in the saffron group (p<0.001). The difference between the two protocols was significant at the endpoint (cycle 4) (t=5.92, df = 48, p<0.001) in the Total Premenstrual Daily Symptoms A significant difference between cycles 3 and 4 was observed in the saffron group (p<0.001). The difference between the two protocols was significant at the endpoint (cycle 4) (t=8.99, df = 48, p<0.001) in Hamilton Depression Rating Scale Rating Scale	Appetite changes, Headache, Sedation, Nausea, Hypomania (None of adverse effects were severe)	Double-blind	Daily Symptom Report for PMS Hamilton Depression Rating Scale	6%	Yes	N=23	N=24	placebo capsule	Saffron capsule 30 mg	20 to 45	8	RCT	Agha-Hosseini et al. (2007), Iran	2
In total, intervention and control groups had significant differences in terms of changes in the mean severity of PMS over time (p<0.001).	increased appetite, loss of appetite, sedation, nausea, headache, and euphoria	triple-blind	the simultaneous determination of stress, anxiousness, and depression scale (DASS21) shortened premenstrual assessment form (SPAF)	11%	Yes	N=39	N=39	Placebo capsules	saffron capsules, 30-mg	18 to 35	8	RCT	Pirdadeh Beiranvand et al. (2015), Iran	3
After intervention total PMS score in curcumin group significantly decreased from 102.06±39.64 to 42.47±16.37 (mean Difference=49.13, 95% CI: 32.48 to 65.78; p<0.0001 ) Whereas this score after intervention in placebo group had not significant difference with before intervention (106.06±44.12 to 91.60±43.56). (mean Difference= 45.14, 95% CI: 27.71 to 62.55; , p=0.058 )	There was no side effects	double-blinded	daily record questionnaire for PMS	10%	Yes	N=31	N=32	Placebo capsules	Curcumin oral gelatin capsules 100 mg	premenopausal	12	RCT	Khayat et al. (2015), Iran	4
Intervention group revealed a significant reduction (p<0.001) in PMS symptoms and also the mean score of PMS intensity reduced at the three consecutive months after the intervention (p=0.001).	Not mentioned	double-blinded	General Health Questionnaire Premenstrual Syndrome Screening Tool (PSST).	0%	Yes	n = 50	n = 50	placebo capsule	Melissa officinalis capsule 1200 mg	16 high school	12	RCT	Akbarzadeh et al. (2015), Iran	5
The mean reduction in severity of PMS score was 6.79 in intervention, and 8.82 in placebo group which did not show any significant difference between the groups (p=0.157). Although Wilcoxon test showed that the severity of PMS score reduced significantly in both groups after the intervention. (In intervention group: Z=3.58, p=0.0001; in placebo group: Z=4.2, p=0.0001) The frequency of reported symptom score decreased from 84.13 in the first menstrual cycle to 56.5 in the fourth cycle in intervention group but this decrease was not significantly different in intervention and placebo groups	Not mentioned	double-blinded	prospective record of the impact and severity of menstrual symptoms (PRISM) calendar	14%	Yes	N=37	N=38	placebo pill	Zataria Pill, 80 mg	18 to 35	8	RCT	Sodouri et al. (2013), Iran	6
Wheat germ significantly reduced physical symptoms (63.56%), psychological symptoms (66.30%), and the general score (64.99%). Although the severity of symptoms decreased in both groups, this reduction was more significant in intervention group (p<0.001). On the other hand, physical symptoms decreased only in the intervention (p<0.001) and there was no statistically significant difference in the placebo group.	digestive complications (3 people) Complications were not reported in 95.2% of the wheat germ extract group and 92.9% of the placebo group.	triple-blind	Beck Depression Inventory (BDI) Daily Symptom Record (DSR)	16%	Yes	N=47	N=37	Placebo	capsules of wheat germ extract 400 mg	20 to 45	8	RCT	Ataollahi et al. (2015), Iran	7
Most of the PMS VAS scores were dropped in both groups, however it was more significant in the Vitex agnus group (p<0.0001). Mean Rank of differences of the headache, nervousness, restlessness, depression, breast pain and swelling, swelling and tympani had significantly difference, before and after the study, in both groups and between two groups (p<0.0001).	There was no side effects	double-blind	Penn daily symptom report self-assessment with visual analogue scale (VAS)	0%	Yes	N=66	N=62	40 drops of placebo	40 drops of Vitex agnus	child bearing age	24	RCT	Zamani et al. (2012), Iran	8
There was not a significant difference in the severity of premenstrual syndrome between the E. platyloba and placebo group before the intervention (100.8±22.1 vs. 104.3±19.5). With a significant difference was found between two groups after the intervention [(49.7±23.2 vs. 79.1±28.1), p=0.002].	unpleasant taste, nausea and vomiting	single blind	Daily Record of Severity of Problems form (DRSP)	0%	Yes	N=30	N=30	Placebo, 30 drops	Echinophora platyloba extract 30 drops	18 to 25	8	RCT	Delaram M. (2014), Iran	9
All the symptoms showed a significantly greater improvement with the fennel extract than placebo (p<0.05) except bloating which was unaffected by the treatment.	Visual symptoms in 1 person of intervention, Allergic reactions, Gastric upset, Respiratory symptoms in 3 persons of placebo	double-blind	Daily Record of Severity of Problem Questionnaire (DRSP-Q)	0%	No	n = 30	n = 30	placebo	30 drops of fennel extract	Women ???	12	RCT	Delaram and Heydarnejad. (2011), Iran	10
A significant difference was observed in mean premenstrual mood (p<0.001) and behavioral (p<0.001) symptoms severity of in the intervention group before and after the intervention the difference in mean of mood and behavioral symptoms before the intervention, and one, two, and three months after the intervention in the intervention group was significant (p<0.001).	There was no side effects	Double-blind	provisional diagnosis Form of PMS Day marks registration form	0%	Yes	N=50	N=50	placebo	valerian pills	18 to 35	12	RCT	Behboodi Moghadam et al. (2014), Iran	11
The severity of Symptoms after treatment in both groups significantly decreased (p<0.001) But the severity of symptoms reduction significantly was higher by citrus essence	There was no side effects	Double-blind	Daily registration Symptoms form	0%	Yes	N=40	N=40	10 drops placebo	10 drops of Citrus aurantiun	18 to 35	8	RCT	Ozgoli et al. (2012), Iran	12
The rate of decrease in Severity of PMS Symptoms after taking hypericum perforatum were 46.45% and 18.1% in placebo and there are Significant differences between the two groups in rate of PMS Symptoms reduction(p=0/000)	There was no side effects	Double-blind	Registration form of temporary status of premenstrual syndrome	5%	Yes	N=31	N=35	placebo	60 drops of hypericum perforatum	female Students ???	8	RCT	Pakgohar et al. (2005)	13
Ginger could reduce The overall intensity of PMS and severity of mood, physical and behavioral symptoms significantly (p<0.001)	There was no side effects	Double-blind	temporary diagnosis form of PMS Daily symptom registration form	5%	Yes	N=33	N=33	Placebo capsules	250 mg Ginger capsules	18 to 35	12	RCT	Khaiat et al. (2014), Iran	14
The results showed a reduction of symptoms in treatment group comparing to the placebo. So that the mean severity was reported 23.64 in the Perforan group, and 46.37with (p=0.001) in the placebo group.	There was no side effects	double blinded	temporary diagnosis form of PMS	7%	Yes	N=45	N=48	placebo	Perforan 480 mg	18 to 35	12	RCT	KHeirkhah et al. (2013), Iran	15
The severity of psychological, physical, behavioral and overall symptoms of premenstrual syndrome 1-2 months after taking intervention in the valerian group was significantly lower than the control group (p<0.001)	Not mentioned	double blinded	Demographic and menstrual history Dickerson questionnaire	0%	Yes	N=60	N=60	placebo capsules	Valerian Officinalis capsules 530 mg	female students ???	8	RCT	Kamranpour et al. (2015), Iran	16
The mean scores of PMS physical symptoms (p<0.001) , Psychological symptoms (p<0.05) and PMS duration (p<0.01) Between two groups had a significant difference during the second and third cycle	Not mentioned	double blinded	Rossignol standard questionnaire Recorded daily symptoms of menstruation	15%	Yes	N=31	N=30	placebo	Vitagnus , 44 drops	female students ???	12	RCT	Mousavi et al. (2014), Iran	17
The PMS improved significantly in both intervention groups during the first and the second month after the intervention. In the Vitexagnus and Flaxseed groups, the mean total PMS score were significantly lower than that in the control group at the first months. In the second month There was no significant difference between the Vitexagnus and Flaxseed groups in terms of the PMS score. In comparison of Flaxseed and Vitexagnus groups after intervention ( mean difference = -0.1; 95% CI:-2.4-0.3; p=0.187)	In flaxseed Group diarrhea (2 cases) In vita gnus Group nausea (1 cases)	triple-blind	The Shortened Premenstrual Assessment Form: PAF	1%	Yes	N=52	N=52 N=53	The third group placebo of vitagnus and Package containing 25 grams of wheat flour	Flax seed powder25 g daily Plus placebo pills ofVitex agnus castus Vitex agnus castus pills Plus placebo of flaxseed	18 to 45	8	RCT	Mirghafourvand et al. (2015), Iran	18

**Table 2:** Methodological assessment of study quality

No	Studies	Criteria for methodological assessment of study quality						
		A	B	C	D		E	F
					1	2		
1	Sharifi et al. (2014)	+	+	-	+	Not mentioned	+	+
2	Agha-Hosseini et al. (2007)	+	+	+	+ less than 20%	+	+	+
3	Khayat et al. (2015)	+	+	+	+ less than 20%	Not mentioned	+	+
4	Akbarzadeh et al. (2015)	+	+	+	-	Not mentioned	+	+
5	Sodouri et al. (2013)	+	+	+	+ less than 20%	Not mentioned	+	+
6	Pirdadeh Beiranvand et al. (2015)	+	+	+	+ less than 20%	-	+	+
7	Ataollahi et al.(2015)	+	+	+	+ less than 20%	-	+	+
8	Zamani et al.(2011)	+	+	+	-	-	+	+
9	Delaram (2014)	+	+	+	-	Not mentioned	+	+
10	Ozgoli et al.(2011)	+	+	+	-	Not mentioned	+	+
*11*	*KHeirkhah* et al. (2013)	+	+	+	+ less than 20%	Not mentioned	+	+
12	Mousavi* et al. (2015)*	+	+	+	+ less than 20%	-	+	+
13	Pakgohar et al. (2006)	Not mentioned	+	+	+ less than 20%	Not mentioned	+	+
14	Kamranpour et al. (2015)	+	+	+	-	Not mentioned	+	+
15	Mirghafourvand et al.(2016)	+	+	+	+ less than 20%	Not mentioned	+	+
16	Behboodi Moghadam et al. (2014)	+	+	+	-	Not mentioned	+	+
17	Delaram & Heydarnejad (2011)	Not mentioned	+	+	-	Not mentioned	+	+
18	Khaiat et al. (2014)	+	+	+	+ less than 20%	Not mentioned	+	+

**The effect of curcumin on PMS**

Khayat et al., found significant reductions in physical, behavioral and mood scores of curcumin group after intervention compared to before intervention (41.4±21.6 -18.13±10.92, p<0.0001), (22.8±17.4 -9.21±5.49, p<0.0001), (37.8±18.3 -15.13±7.48, p<0.0001), respectively). In the placebo group, mean of physical score after intervention significantly decreased from 46.7±26.8 to 38.50±20.27 (p=0.0425), whereas mean of behavioral and mood scores after intervention were not significantly different from before intervention scores ((24.4±19.4 to23.14±17.27, p=0.3544) and (34.8±22.4 to 33.85±18.04, p=0.4006), respectively). Collectively, in curcumin group, total PMS score significantly decreased from 102.06±39.64 to 42.47±16.37 (p<0.0001), whereas this score after intervention in placebo group did not significantly differ compared to before intervention (106.06±44.12 to 91.60±43.56, p=0.058) (Khayat et al., 2015 ▶).

**The effect of** ***Melissa officinalis*** **on PMS**

Study by Akbarzadeh et al., showed progressive decline in the mean of PMS symptoms (physical, Psychological and Social) in *M. officinalis*-treated group (42.56±15.73, 30.72±13.24, 30.2±12.08, 13.90±10.22) at baseline, as well as 4, 8 and 12 weeks after trial and the differences were statistically significant (p<0.0001). Using paired t-test, it was demonstrated that the force of physical, psychological and social symptoms significantly diminished in the intervention group. However, no significant difference was found in the control group in this regard (p<0.001). No side effects related to *M. officinalis* plant were noted in this study (Akbarzadeh et al., 2015 ▶).

**The effect of** ***Zataria multiflora*** **on PMS**

Sodouri et al., reported a non-significant difference between Intervention and Placebo groups in terms of reductions in the mean of PMS severity score (p=0.157). Wilcoxon test demonstrated that the severity of PMS score decreased significantly in both groups after the intervention (In intervention group: Z=3.58, p=0.0001; in placebo group: Z=4.2, p=0.0001). The repeated-measure analysis of variance revealed that the recurrence of symptom score markedly diminished in the menstrual cycles; however, this decline was not significantly different in intervention and placebo groups (p=0.35) (Sodouri et al., 2013 ▶).

**Table 3:** Herbs names in different language

**Persian name**	**English name**	**Scientific name**	**The modes of action**
**Baboone**	Chamomile	Matricaria *chamomila*	anti-inflammatory, antioxidant, analgesic, antineoplastic, anti-anxiety, and digestive effect
**Zafran **	Saffron	*Crocus sativus *	Serotonergic, antidepressant, antispasmodic
**Zardchoobe**	Turmeric	*Curcuma longa*, Curcumin	antidepressant, anti-inflammatory, antioxidant, anti-carcinogenic, anti-arthritic, thrombo suppressive, anti-microbial, and Hypoglycemic reduces prostaglandins synthesis modulation of neurotransmitters levels
**Badranjbooie**	Lemon balm	*Melissa officinalis*	anti-depression, sedative and improve cognitive function
**Avishan Shirazi**	Zataria multiflora	*Thymus vulgaris*	antibacterial agent, anti-dyspepsia, inhibit mediators of inflammatory reactions, antioxidant, antispasmodic, analgesic and antiseptic
**Gandom**	Wheat Germ Extract	Triticum spp	contains magnesium, zinc, calcium, selenium, sodium, potassium, phosphorus, chromium, antioxidants including beta-carotene (for vitamin A), vitamin E, vitamin C, vitamin B12, vitamin B6, thiamin, riboflavin, niacin, folic acid, iron, amino acids, and enzymes so has a high dietary and medicinal value
**5 angosht**	chaste berry	*Vitex agnus* *castus* berries	regulation of stress, induced prolactin discharge via dopamine, Binding to opioid receptors, endorphins, and neuroactive flavonoids may also have a role
**Khousharizeh** **Or Tigh Touragh**	prickly parsnip	*Echinophora platyloba*	antibacterial components, anti-spasmodic effects, anti-fungal effects
** Raziane**	Fennel	*Foeniculum vulgare*	relieve painful menstruation , inhibitory effect on the production of oxytocin and Prostaglandin E2 (PGE2), the mode of action of fennel appears to be hormonal.
**Katan**	Flax Seed	*Linum usitatissimum* L.	Contain :Fiber, manganese, vitamin B1, alpha-linolenic fatty acid, omega-3 and phytoestrogen, antioxidant, Anti-inflammatory
**Sonbolettib**	Valerian	*Valeriana officinalis*	Sedative effects Inhibition of contractions Antispasmodic Antidepressants
**porteghal**	Sweet – Orange tree	*Citrus sinensis*	Stimulation of the CNS and mood-enhancing effects, sedation, Antispasmodic anti-inflammatoire Antidepressants
**Gole raei or alaf chai**	St. John's Wort	*Hypericum perforatum* or Perforan	Antidepressants Prevention of Serotonin reuptake and Amino oxidase activity
**zanjabil**	Ginger	*Zingibar officinale*	Effect on prostaglandin system, Anti-nausea, vomiting, dizziness, Prostaglandin inhibitor, Headache relief, Anti-rheumatoid

**The effect of wheat germ extract on PMS**

Ataollahi et al. found that mean reduction in physical symptoms (headache, breast tenderness, acne, swelling, bloating and palpitations) in intervention and the placebo groups was 63.56% and 14.35%, in psychological symptoms (irritability, tension, sleep problems, mood swings, food cravings, wish to be alone, depression, forgetfulness, anxiety, poor concentration, crying, depression, suicide, decreased libido and fatigue) was 66.30% and 27.43%, and in general symptoms was 64.99% and 21.05%, respectively.

The severity of the general and psychological symptoms diminished in two groups, yet this reduction was significantly more pronounced in the wheat germ extract group (p<0.001). The decrease in severity of physical symptoms was statistically significant just in the wheat germ extract group (p<0.001), and there was not a statistically significant difference in the placebo group. A considerable reduction in severity of symptoms following two months of treatment observed in fatigue (85.32%), irritability (84.80%), palpitation (80.24%), irritation (80.24%), breast tenderness (79.71%), headache (76.52%), sleep problems (73.79%), increased appetite (73.12%), acne (70.40%), mood swings (69.64%), food cravings (68.71%), wish to be alone (68.10%), depression (61.64%), forgetfulness (59.15%), anxiety (58.94%), poor concentration (56.72%), crying (44.53%) and breast swelling (25.72%). The number of consumed painkillers remarkably reduced after intervention in these cases (p<0.001); Complications were not announced in 95.2% of the wheat germ extract group and 92.9% of the placebo group (Ataollahi et al., 2015).

**The effect of** ***Vitex agnus-castus*** **on PMS**

*Vitex agnus-castus vs. placebo*

Three trials (Zamani et al., 2012 ▶, Moosavi et al., 2014 ▶, Mirghafourvand et al., 2015 ▶) investigated the effect of *Vitex agnus-castus* on PMS. Zamani et al. reported a reduction in a large portion of the PMS VAS scores in two groups, in any case it was more significant in the *Vitex agnus* group (p<0.0001). Mann-Whitney test indicated that Mean Rank of differences in the headache, nervousness, restlessness, depression, breast pain and swelling, swelling and tympani was significantly different between pre- and post-treatment in both groups and between two groups (p<0.0001). No adverse effect was addressed. Another trial performed by Mousavi et al. showed that mean scores of PMS physical symptoms (p<0. 001) and PMS psychological symptoms (p<0. 05) were significantly different between two groups in the second and third cycles of treatment. The mean duration of PMS was significantly different between the two groups (p<0. 01). Some adverse effects were reported in two subjects of intervention group including itching, rash and dizziness and in one subject of the control group, stomachache and diarrhea were seen.

***Vitex agnus castus vs. Flaxseed***

Three groups was investigated in Mirghafourvand et al. study and finding showed; PMS was significantly improved in both intervention groups during the first and the second month after the treatment. In the *Vitex agnus* and Flaxseed groups, the mean total PMS scores were significantly lower than those of the control group that received placebo after the treatement (adjusted mean difference: -3.3 (95% CI: -4.0 to - 2.1); -4.3 (-5.5 to -3.0), respectively). In the second month after the intervention, the mean total PMS score was -5.8 (-7.0 to -4.7) in the *Vitex agnus* group and -6.6 (-8.1 to -5.7) in the Flaxseed group. There was no significant difference between the *Vitex agnus* and Flaxseed groups in terms of the PMS score. Only one person in *Vitex agnus* group reported mild nausea.

**The effect of** ***Echinophora platyloba*** **on PMS**

Delaram reported there was no significant difference in severity of PMS between two groups before the intervention, while a significant difference was observed after the intervention (p=0.002). Also, the severity of anxiety and depression in intervention groups was significantly lower than the placebo group. The participants in the *E. platyloba* group reported reductions in the severity of symptoms, with the most marked reduction being seen in depression (p=0.029) and anxiety (p<0.001) symptoms. Two participants complained of an unpleasant taste, nausea and vomiting (Delaram, 2014).

**The effect of Fennel (** ***Foeniculum vulgare*** **) on PMS**

Delaram and Heydarnejad found a significantly greater improvement in combined symptoms (stress, depression, excitement, somatic and cluster symptoms) in the treatment group than placebo of the fennel (p<0.05) except for the bloating which was unaffected by the treatment. The reductions in the percentages of all symptom scores in the treatment group were significantly more pronounced than the placebo group. There were few adverse events such as visual symptoms related to consumption of fennel extract in one person (Delaram and Heydarnejad, 2011)

**The effect of valerian root extract on PMS**

Two trials (Moghadam et al., 2014 ▶, Kamranpur et al., 2015 ▶) determined the impact of valerian on PMS. In one trial done by Kamran pour et al., according to Friedman test, there was a significant difference in the severity of mood, physical and behavioral symptoms among baseline, 4 weeks and 8 weeks after taking valerian in both intervention and placebo (50 mg starch) groups (p<0.001) and this difference was more pronounced in experimental group than placebo.

In another study done by Behboodi Moghadam et al., results showed a significant difference in mean severity of premenstrual mood (p<0.001) and behavioral (p<0.001) symptoms in the valerian group compared to placebo, before and after the intervention. Moreover, the difference in mean score of mood and behavioral symptoms was significant among baseline, and one, two, and three months after the intervention in treatment group (p<0.001). Nevertheless, this difference was insignificant in the control group.

**The effect of orange peel essence (** ***Citrus sinensis*** **) on PMS**

In one trial done by Ozgoli et al., the rates of reduction in severity of PMS symptoms were 46.08% and 14.21% after using the essence of citrus, and the placebo (water and sugar), respectively. There was a significant difference between two groups regarding the rate of reductions in severity of PMS symptoms (p<0/001). Also, reductions in severity of physical symptoms were 24.30% in the case group and 5% in the placebo group. The rate of reduction in severity of psychological symptoms were 21.78% in the case group and 9.21% in the placebo group (p<0/001). Moreover, 94.7 % in treatment group and 100% in placebo group did not report any side effects (Ozgoli et al., 2012 ▶)

**The effect of** ***Hypericum perforatum*** **on PMS**

Two trials (Pak Gohar et al., 2006 ▶; Kheirkhah et al., 2013 ▶) checked the effect of *Hypericum perforatum* (perforan) on the PMS. Kheirkhah et al. found a decrease in symptoms in treatment group comparing to the placebo group. In this regard, the mean of severity was 23.64 in the perforan-treated group, and 46.37 (p=0.001) in the placebo group. There were no side effects in both groups. In another trial done by Pak Gohar et al., the rates of reductions in severity of PMS symptoms were 46.45% in group treated with *H. perforatum* and 18.1% in placebo group. Thus, the rates of reduction in severity of PMS symptoms in intervention groups were significantly more marked than the placebo group (p=0.000).

**The effect of ginger on PMS**

In one trial done by Khayat et al., there was a significant difference between the two groups in terms of overall severity of PMS and severity of physical, mood and behavioral symptoms after intervention (p<0.001). Ginger significantly reduced overall severity of PMS and severity of physical, mood and behavioral symptoms (p<0.001). One subject in the intervention group experienced gastrointestinal side effects (Khayat et al., 2015 ▶).

**The effect of flaxseed on PMS**

Mirghafourvand et al. randomly divided patients into three groups of Flaxseed, *Vitex agnus* and placebo. According to repeated measurement ANOVAs, the PMS was significantly improved in both intervention groups during the first and the second month after the treatment. In the *Vitex agnus* and Flaxseed groups, the mean total PMS scores were significantly lower in control group that received placebo (adjusted mean difference: -3.3 (95% CI: -4.0 to -2.1); -4.3 (-5.5 to 3.0), respectively). In the second month after the intervention, the mean total PMS score was -5.8 (-7.0 to -4.7) in the *Vitex agnus* group and -6.6 (-8.1 to -5.7) in the Flaxseed group. There was no significant difference between the *Vitex agnus* and Flaxseed groups in terms of the PMS score. One person in the Flaxseed group reported diarrhea and one person in *Vitex agnus* group reported nausea (Mirghafourvand et al., 2015).

## Discussion

Herbal medicines have been widely utilized for the treatment of numerous diseases including premenstrual syndrome (PMS). However, limited systematic reviews have been done on Iranian herbal medicines in terms of their effectiveness and safety. To the best of our knowledge, this is the first systematic review about therapeutic effect of Iranian herbal medicine on PMS. Overall, studies have shown that *Vitex agnuscastus, Hypericum perforatum, Matricaria chamomilla, saffron, Curcumin*, *Melissa officinalis*, *Zataria multiflora,* *Wheat Germ Extract, Echinophora platyloba, Foeniculum vulgare, Valerian root extract, Citrus sinensis, Zingiber officinale *and *Flax seed *may alleviate symptom of PMS (PakGohar et al., 2006; Agha‐Hosseini et al., 2008; Delaram and Heydarnejad, 2011; Ozgoli et al., 2012, Zamani et al., 2012, Beydokhti et al., 2013; Kheirkhah et al., 2013, Sodouri et al., 2013; Delaram, 2014, Khaiat et al., 2014; Moghadam et al., 2014; Moosavi et al., 2014; Sharifi et al., 2014; Akbarzadeh et al., 2015; Ataollahi et al., 2015; Beiranvand et al., 2015; Kamranpur et al., 2015; Khayat et al., 2015; Mirghafourvand et al., 2015; Saki et al., 2015) .

The current evidence underlines the essential role of serotonergic system during the luteal phase in women experiencing PMS. Likewise, the effect of sexual hormones on the uptake, binding, turnover, and transportation of serotonin has been reported (Halbreich et al., 2003 ▶).

It has been suggested that dysregulation of the serotonergic system, specifically alterations in serotonin activity, is responsible for most PMS symptoms (Andrus, 2001 ▶). Reviewing these studies indicated that most of these Iranian herbal plants possess antidepressant effects through a serotonergic mechanism and their anti-inflammatory effects can reduce general symptoms of PMS, especially the psychological signs. Modes of action of these herbal medicines are shown in Table 3. In this way, the present investigation reports that the importance of serotonergic operators in the treatment of PMS (Dimmock et al., 2000 ▶). our research evaluated modes of action of herbal medicines on PMS and in clinical practices, chemical drugs such as mefenamic acid and fluoxetine are effective in reduction of PMS symptoms through mechanisms mentioned above (Brown et al., 2009 ▶). Review showed that in the control group, there was also a decline in the severity of symptoms. Several factors may be involved in placebo response, including doctor-patient relationship, patients' positive or negative expectations of treatment, cultural factors like patients' perception of colors, forms, and drug names, along with their experience and perception of fate and faith (Moerman, 2000, Del Giorno et al., 2010). This systematic review showed that placebo had a slight effect on alleviating PMS symptoms. One possible explanation for this can be cultural difference between participants. Since the investigations in assessing the treatments of PMS without the utilization of control group and placebo are under serious questions. Indeed, even in randomized clinical trials, the placebo reactions must be reduced. Follow-up of women for more than three menstrual cycles can confine the placebo responses in PMS interventional studies (Zomorodian et al., 2011 ▶).

In the current systematic review, the length of treatments (three menstrual cycles) might not be adequate to minimize the placebo impact. Except four study (Sodouri et al., 2013 ▶, Moosavi et al., 2014 ▶, Akbarzadeh et al., 2015 ▶, Kamranpur et al., 2015 ▶) which did not mention the side effects resulting from the use of medicinal plants, in other studies, side effects were not observed or had low-intensity and observed in less than 20% of the cases (Table 2). Some studies showed that side effects of herbal products are less common than synthetic drugs (Younesi, 2014 ▶; Karimi etal.,2015 ▶)**. **Since, chemical drugs have side effects So, medicinal plants can be an alternative for chemical drugs in PMS treatment. However, medicinal plants should be recommended by physicians when an individual suffers from a special disease or is using other drugs.

Many studies have shown beneficial effects of herbal medicine in decreasing hot flashes. Future studies can compare the effectiveness of Iranian herbal medicines with native plants of other countries and with other methods suggested by alternative medicines for treatment of PMS. Further studies are required to compare side effects (safety) and efficacy of herbal remedies with those of the synthetic drugs. Trials should be comprehensively addressed and investigated in detail as completely as possible, conforming to the CONSORT guidelines (Dworkin et al., 2010 ▶) Also, Future trials should construct their plan with respect to CONSORT guideline in order to increase the quality of the trials. Also, further investigations with larger sample size, among females from different socioeconomic levels of the community, using different doses of herbal medicine for longer periods of time as well as studies without using a placebo, are suggested to achieve more definitive results about the effectiveness and safety of herbal medicine for alleviating PMS symptoms**.**

Although we believe our search was comprehensive, publication bias as a problem in all medical research, may have affected our evaluations (Easterbrook et al., 1991 ▶) and this issue may be intensified in alternative medicine research (Ernst and Pittler, 1997 ▶). It has also been argued that a narrative summary is susceptible to bias, subjectivity, and limited in the absence of an effect size (Graham, 1995 ▶). In order to minimize this possibility, two reviewers discussed the study findings and quality indicators in depth, as well as the potential impact of methodological shortcomings, resolving discrepancies with the third reviewer if necessary. The weak methodology of many studies included in our systematic review can be one of the potential limitations of this study. Small sample sizes, inadequate treatment allocation, unclear blinding method and unmentioned randomization technique may influence the validity of the results.

This research demonstrated the efficacy of herbal medicines in alleviating PMS symptom (Adib-Hajbaghery and Hoseinian, 2014 ▶, Menati et al., 2014 ▶). Therefore, herbal medicines can be considered as an appropriate alterative for women experiencing PMS
